# 3D volumetric analysis of upper airway changes following orthognathic surgery

**DOI:** 10.1186/s13005-025-00557-y

**Published:** 2025-11-24

**Authors:** Lukas B. Seifert, Stephani V. Cleanthous, Michelle Klos, Sarah Bühling, Sead Abazi, Britt-Isabelle Berg, Florian M. Thieringer, Robert Sader

**Affiliations:** 1 Department of Oral and Cranio-Maxillofacial Surgery, University Hospital Basel, University of Basel, Basel, Switzerland; 2 Medical Additive Manufacturing Research Group (Swiss MAM), Department of Biomedical Engineering, University of Basel, Basel, Switzerland; 3 Department of Oral, Cranio-Maxillofacial and Facial Plastic Surgery, University Hospital Frankfurt, Goethe University, Frankfurt am Main, Germany; 4 Department of Orthodontics, Polyclinic of Orthodontics, University Hospital Frankfurt, Goethe University, Frankfurt am Main, Germany

**Keywords:** Orthognathic surgery, Airway volume, Cone beam computed tomography, Mandibular advancement, Obstructive sleep apnea, Virtual planning

## Abstract

**Background:**

Orthognathic surgery is frequently performed to correct dentofacial deformities and is known to alter the upper airway morphology. This study aimed to analyze volumetric changes in the upper airway following mono- and bimaxillary surgery using virtual surgical planning, with a specific focus on the minimal cross-sectional area (mCSA) and total upper airway volume (TUAV).

**Methods:**

In this retrospective study, 81 patients with Angle Class II or III malocclusions underwent either mandibular advancement (MA), maxillomandibular advancement (MMA), mandibular setback, or a combination of mandibular setback with maxillary advancement. Surgical planning was performed using IPS CaseDesigner® software. Pre- and postoperative CBCT scans were analyzed to assess changes in mCSA and TUAV. Displacement distances were measured for both jaws, and statistical analysis was performed using paired t-tests and Pearson correlation.

**Results:**

MA and MMA in Class II patients resulted in significant increases in mCSA (*p* = 0.0048 and *p* = 0.0005, respectively) and TUAV (*p* = 0.0346 and *p* = 0.01, respectively). In contrast, mandibular setback in Class III patients showed non-significant decreases in airway parameters, while bimaxillary surgery produced slight, non-significant increases. A mandibular setback of less than 4.05 mm was not associated with a relevant reduction in airway volume.

**Conclusion:**

MA and MMA significantly increase upper airway dimensions and are effective treatment strategies for oropharyngeal airway deficiencies in Class II patients. Minor mandibular setbacks (< 4.05 mm), especially when combined with maxillary advancement, appear to preserve airway volume in Class III patients. These findings highlight the importance of individualized virtual planning to optimize both functional and aesthetic outcomes, while recognizing the limitations of using static CBCT imaging to assess dynamic airway function.

## Introduction

Orthognathic surgery constitutes the treatment of craniofacial deformities by surgically repositioning the upper and/or lower jaw, which can alter the shape and morphology of the upper airway [1, 2]. Maxillary and/or mandibular surgical adjustments may lead to changes in the volume and configuration of the oral and nasal cavities, depending on the magnitude and direction of correction [3]. Accordingly, orthognathic surgery can be applied as a treatment approach for obstructive sleep apnea (OSA) [4–7], a condition characterized by partial or complete pharyngeal airway collapse with symptoms such as loud snoring, fragmented sleep, and impaired cognition [8–10]. However, while such procedures can affect airway dimensions, functional outcomes such as improvements in sleep quality or OSA are multifactorial and cannot be predicted solely on the basis of static volumetric changes measured with CBCT.

The pharyngeal upper airway volume requires careful consideration in orthognathic surgery because of its association with airflow dynamics and potential implications for sleep-disordered breathing [11]. Airway narrowing following certain surgical movements may increase airflow resistance and potentially predispose patients to sleep apnea [12, 13]. For instance, mandibular setback to correct mandibular prognathism (Class III malocclusion) can reduce retrolingual space and thereby diminish oro- and hypopharyngeal dimensions. Alternatively, bimaxillary surgery, which combines mandibular setback with maxillary advancement, may help mitigate airway compromise. The objectives of maxillary advancement in patients with Class III dentofacial deformity are to improve dental occlusion and facial symmetry, while supporting airway patency and reducing the extent of mandibular setback [14–16].

Conversely, mandibular retrognathism (Class II malocclusion) is commonly treated with mandibular advancement (MA) surgery, as it may increase intramaxillary space, and retropalatal and retrolingual volumes [16], proving to be an effective surgical approach to treating oropharyngeal airway deficiencies [17]. Maxillo-mandibular advancement (MMA) is another orthognathic surgical approach used to treat oropharyngeal airway deficiencies such as OSAS, especially in patients who are not able to maintain continuous positive airway pressure and who do not respond to other treatments [17, 18]. Nonetheless, these procedures cannot ensure long-term airway stability or prevention of OSA, which is known to occur across all skeletal malocclusion classes and is not exclusively linked to reduced static airway dimensions.

The purpose of this study is to evaluate changes in the minimal cross-sectional area and total upper airway volume in skeletal Class II and Class III patients undergoing different surgical movements (mandibular advancement/maxillomandibular advancement vs. mandibular setback/bimaxillary surgery) within a standardized virtual planning workflow, and to explore to which mandibular setback can be performed without causing clinically relevant airway restrictions.

## Materials and methods

**Fig. 1:**
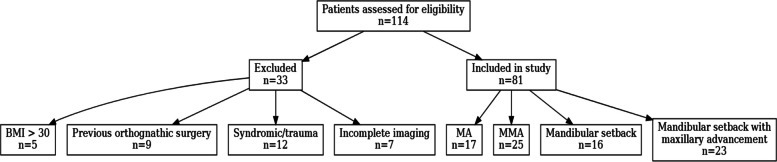
Strobe flow diagram depicting inclusion/exclusion and group allocation

This retrospective study was conducted on data collected from 81 patients who were treated at the Department of Oral and Maxillofacial Surgery, Goethe University Clinic, Frankfurt from 01 January 2021 to 31 August 2023. The inclusion and exclusion process, as well as group allocation, is summarized in the STROBE flow diagram (Fig. 1). Inclusion criteria were adult subjects of both genders (female, male), with an Angle Class II or III malocclusion, who underwent mandibular or bimaxillary orthognathic surgery. Subjects with a BMI greater than 30 kg/m^2^, previous orthognathic surgery, medical history of facial trauma, congenital syndromes, or malformations, were excluded from the study.

### Surgical treatment planning

**Fig. 2:**
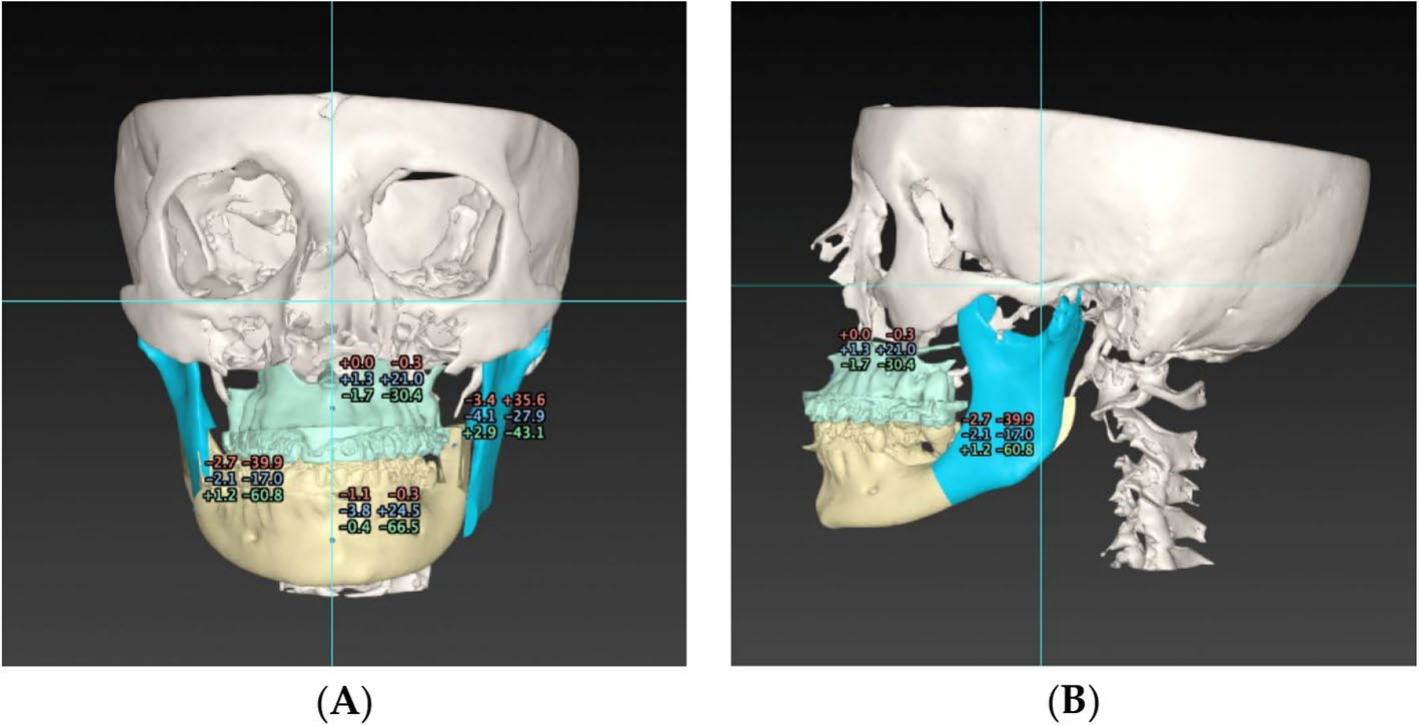
Surgical treatment planning with IPS CaseDesigner ® software for Angle Class III malocclusion: displacement distances of the maxilla and mandible from the front (**A**) and side (**B**)

Each surgical treatment plan followed a digital workflow (Fig. 2). 3D intraoral scans were taken using the 3Shape TRIOS 4 scanner (3Shape, Copenhagen K, Denmark), uploaded into the 3Shape Unite Software (Version 24.1, 3Shape, Copenhagen K, Denmark), and saved as STL files. The pre-operative cone beam computed tomography (CBCT) scans were uploaded into the IPS CaseDesigner ® (Version 2.3.5.2, KLS Martin Group, Tuttlingen, Germany) to accurately represent the patient’s anatomical components, e.g., skull, teeth, and soft tissues. The intraoral scans were then incorporated to further enhance details in dental structures. This software produced a 3D reproduction of the craniofacial anatomy encompassing the maxillae, mandibles, and upper airways. Cephalometric analyses were performed to identify skeletal discrepancies and dental malocclusions, which were corrected via simulation. The total upper airway volume (TUAV) and minimal cross-section area (mCSA) measurements were assessed to reflect the changes that surgery could have on the airway. The Le Fort I osteotomy (LFI) and bilateral sagittal split osteotomy (BSSO) were visualized to evaluate bone repositioning, thereby improving the accuracy of therapeutic movements such as maxillary and mandibular advancements, setbacks, rotations, and correction of asymmetry.

After the surgical plan was developed, the software produced interocclusal splints which were then transferred into a physical model using 3D printing (Organical 3D print X10, Organical CAD/CAM GmbH, Berlin, Germany). The splints were used during the surgeries to transfer the planned movements of the maxilla and mandible into the situs. Postoperative imaging was performed after tissue swelling had subsided (6 weeks after surgery). These images were compared with the pre-surgical images to assess the accuracy and effectiveness of the surgery.

### Orthognathic surgery

BSSO was carried out with a piezoelectric surgical device and a Lindemann burr, while LFI osteotomies were performed with a surgical microsaw. Both procedures were achieved via an intraoral approach. After mobilizing and moving the mandible into proper position, fixation was achieved using two mini plates with monocortical screws on each side. Similarly, the LFI for maxillary advancement was stabilized using two osteosynthesis plates on each side. There were no other additional surgical procedures, such as genioplasty, bone grafts, or rhinoplasty, performed on the patients. Orthognathic surgery was indicated in patients with skeletal dysgnathia (Class II and III, and asymmetries) with resulting dysfunction in the stomatognathic system. Another reason for MMA was the presence of severe OSAS symptoms. Patients with an Angle Class II malocclusion were treated either with a single MA or a bimaxillary advancement. Patients with an Angle Class III malocclusion underwent a single mandibular setback, or a mandibular setback combined with a maxillary advancement. The orthodontics-first approach was used in all patients, entailing orthodontic treatment both pre-and postoperatively. The required surgical movements (e.g., monomaxillary or bimaxillary surgery, displacement distance, etc.) were decided according to dental occlusion, cephalometry, CBCT image, and facial analysis, with additional individualistic aesthetic considerations for each patient.

### Image acquisition and analysis

All patients underwent a CBCT (ProMax 3D Max ProFace, Planmeca Oy, Helsinki, Finland) examination for assessment of upper airway volume and skeletal changes within a month before surgery (T0), and 6 weeks after surgery (T1) under the same conditions. Postoperative CBCT scans were routinely acquired at 6 weeks after surgery, once the majority of soft-tissue swelling had subsided, as part of the standardized follow-up protocol of the Department of Oral and Maxillofacial Surgery at Goethe University Frankfurt. Each patient sat in an upright position with the head in a neutral position, and teeth in slight contact, while the maxillofacial region was scanned for 18–26 seconds using a CBCT field view of 23 cm x 26 cm, anode voltage of 54–96 kVp, and anode current of 1–12 mA. CBCT data were converted into a DICOM file format and then imported into the surgical planning software IPS CaseDesigner ®.

### Airway analysis

**Fig. 3:**
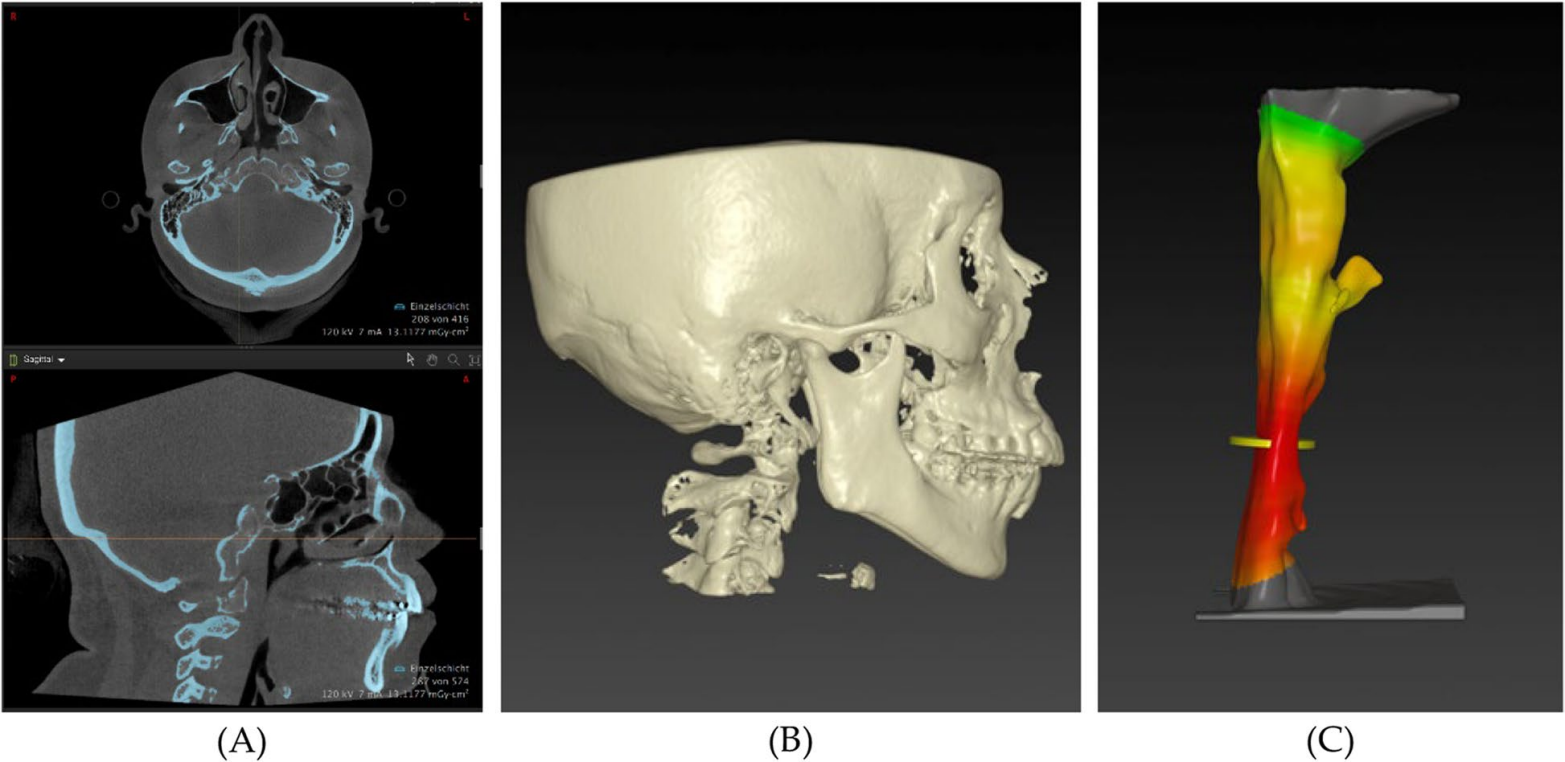
Group 2, Angle Class II malocclusion in the preoperative image: **A** pre-op soft tissue and airway representation, **B** pre-op skeletal representation, **C** pre-op 3D representation of TUAV

**Fig. 4:**
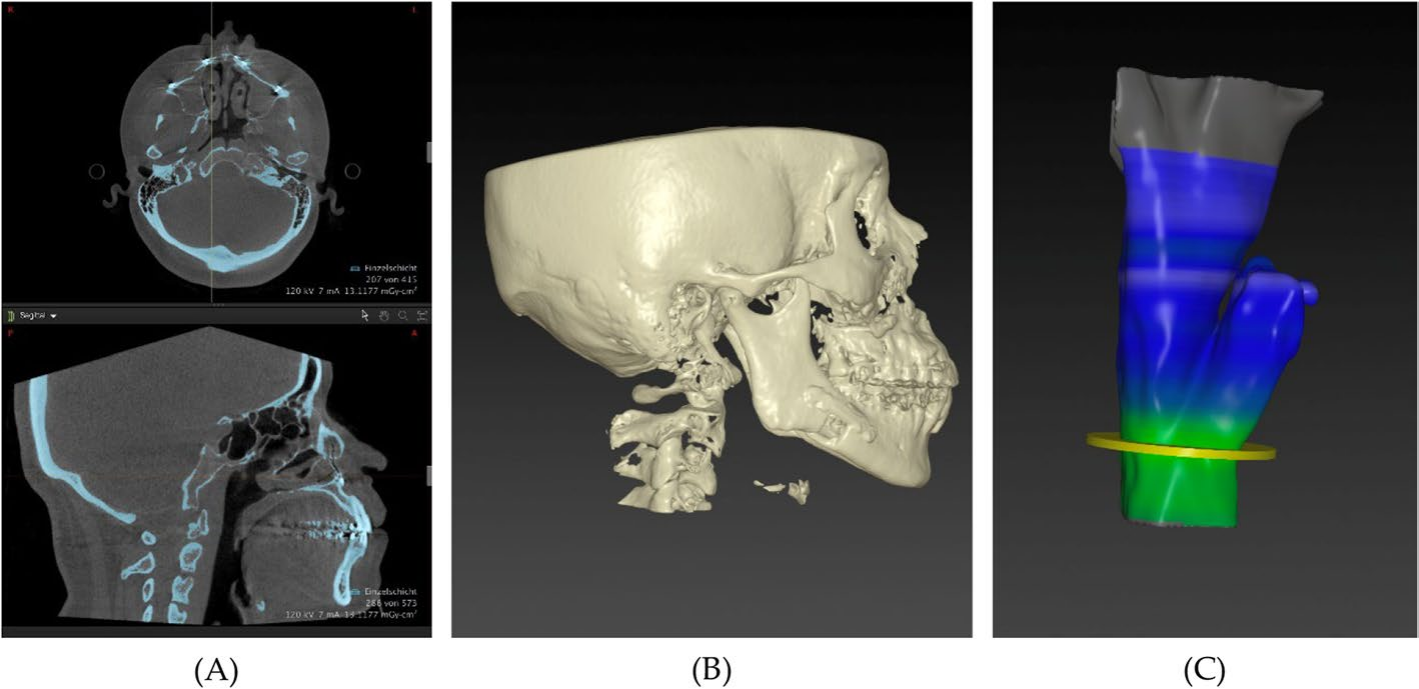
Group 2, Angle Class II malocclusion postoperative image: **A** post-op soft tissue and airway representation, **B** post-op skeletal representation, **C** post-op 3D representation of TUAV

**Fig. 5:**
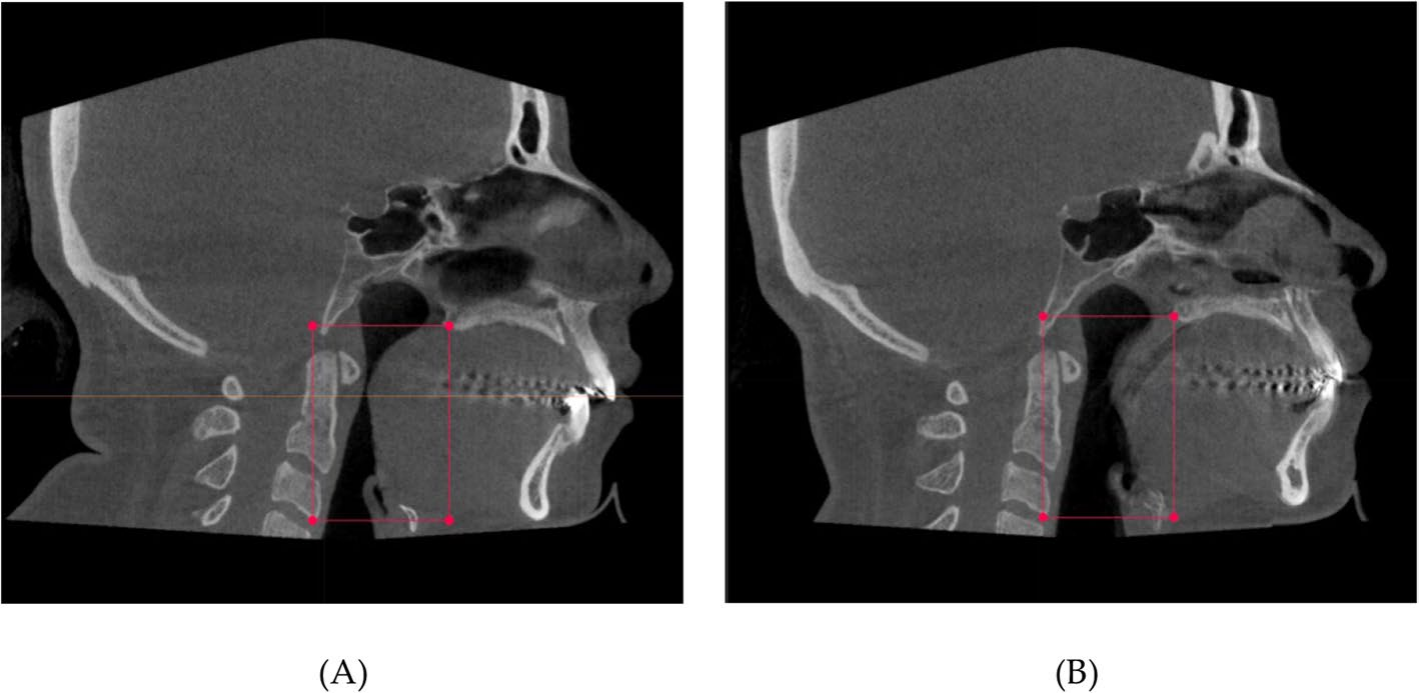
Group 2, Angle Class II malocclusion in the **A** preoperative CBCT image and the **B** postoperative CBCT image. Airway segmentation was defined from the posterior nasal spine/choanae (anterior), to the posterior pharyngeal wall (posterior), from the posterior nasal spine (superior) down to the lower edge of C3 (inferior)

All volumetric measurements were performed by a single assessor trained in the standardized IPS CaseDesigner® workflow, applying predefined thresholds and anatomical boundaries; intra- and inter-rater reliability (ICC) was not assessed in this retrospective setting and is acknowledged as a limitation. DICOM data was imported into the IPS CaseDesigner ® to create a new case for each patient. After checking bone and skin thresholds and setting the neutral head position (NHP), the upper airway volumes of each patient could be measured. The IPS CaseDesigner ® algorithm was used to measure the total upper airway volume (TUAV) and the minimal cross-section area (mCSA) of the velopharyngeal airway of each patient using the DICOM images (Figs. 3 and 4). These parameters were measured on pre- and postoperative images and then compared to each other. The landmarks (Fig. 5) used in these analyses were:

- Anterior: posterior nasal spine in the sagittal plane and choanae in the axial plane
- Posterior: posterior wall of the pharynx
- Superior: the level of the posterior nasal spine
- Inferior: the level of the lower edge of the C3 vertebral body

### Mandibular and maxillary displacement distance

**Fig. 6:**
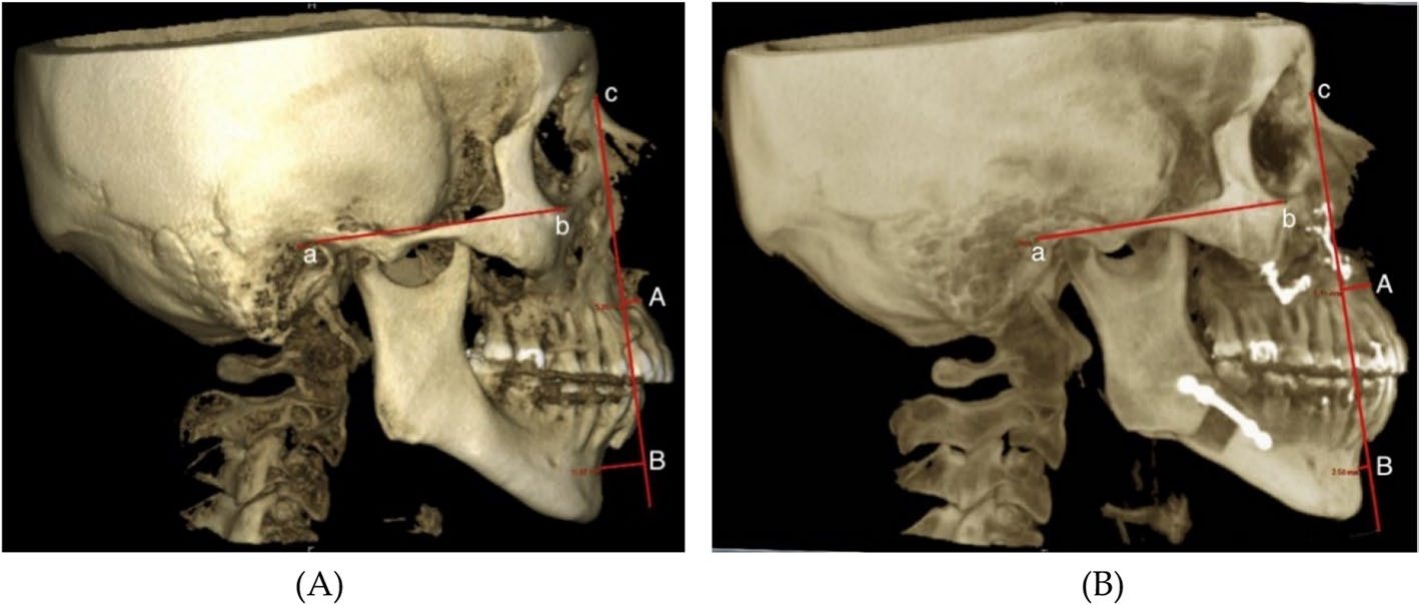
Group 2, Angle Class II malocclusion: **A** preoperative image and **B** postoperative image showing skeletal structures and displacement distances. Measurements were based on the Frankfort horizontal plane, with reference points at the external porus acusticus (a), infraorbital margin (b), a perpendicular line from nasion (c), and distances to A-Point (maxilla) and B-Point (mandible)

The CBCT images of each patient were used to measure the displacement distance of the mandible and maxilla following surgery utilizing measurement tools in Orbis (Agfa HealthCare GmbH, Bonn/Germany). Reproducible reference points were created by first drawing a line perpendicular to the Frankfort horizontal plane, always starting from the most concave point of the nasion, followed by measuring the distances from this line to the most concave points of the mandible (B-Point) and the maxilla (A-Point) (Fig. 6). Representative postoperative 3D renderings and airway segmentations for patients withAngle Class II malocclusion are shown in Figs. 3 and 4. The pre- and postoperative differences of these distances were calculated and documented.

The landmarks used in these analyses were:

- Frankfort horizontal plane:
  - external porus acusticus (a)
  - infraorbital margin (b)
- Perpendicular line to the Frankfort horizontal plane starting from the most concave point of the nasion (c)
  - distances from this line to the most concave points of the mandible (B-Point) and the maxilla (A-Point)

### Statistical analysis

Data analyses were performed with GraphPad Prism ^TM^ v.9.5.1 (GraphPad Software LLC, Dotmatics, USA). The clinical parameters were presented as mean (X), and standard deviation (SD) for the mCSA and TUAV. The pre- and postoperative differences in the mCSA and TUAV were calculated for each surgical group. A paired *t*-test was used to compare the preoperative (T0) and postoperative (T1) mean values of the mCSA and the TUAV change for each surgical group. Pearson’s test was used to correlate total airway change and jaw displacement distance variables. *P*-values less than 0.05 were considered to be statistically significant. Group differences are reported with 95% confidence intervals where variance data were available; for comparisons where paired-difference variances could not be derived from the retrospective dataset, exact *p*-values are provided, and this limitation is explicitly acknowledged. No formal a priori power calculation was conducted, given the retrospective, single-center design. Instead, the study included all eligible consecutive cases from the year 2022 (*n* = 81). This group size is comparable to other CBCT-based airway studies [19] and was considered sufficient to detect moderate within-group changes.

## Results

Eighty-one subjects were included in this study, aged between 18 and 52 years (28.9 ± 8.37). Forty-eight were female (59.3%), and thirty-three were male (40.7%). Forty-two patients were diagnosed with an Angle Class II malocclusion, and thirty-nine patients had an Angle Class III malocclusion. Seventeen subjects (female *n* = 13, male *n* = 4) received a mandibular advancement (MA); twenty-five subjects (female *n* = 16, male *n* = 9) received maxilla-mandibular advancement (MMA), while sixteen subjects (female *n* = 7, male *n* = 9) underwent a mandibular setback and twenty-three (female *n* = 12, male *n* = 11) had a maxillary advancement and mandibular setback.

The Group 1 patients with Angle Class II malocclusion who underwent MA, showed significant post-operative changes in the mCSA (*p* = 0.0048) and the TUAV (*p* = 0.0346). The mCSA increased by an average of 92.26 mm^2^ (± 116.43) and the TUAV increased by an average of 4.91 cm^3^ (± 8.77) (Table 1). The average MA was + 3.84 mm (± 1.83).

**Table 1:** Preoperative and postoperative minimal cross-section area (mCSA) and total upper airway volume (TUAV) in subjects with Angle Class II facial deformity who underwent MA

	mCSA (mm^2^)			TUAV (cm^3^)		
	X	SD	*P* Value	X	SD	*P* Value
T0	254.11	123.23		23.76	8.96	
T1	346.37	145.84	0.0048^*^	28.67	8.20	0.0346^*^
Difference	(+)92.26	± 116.43		(+)4.91	± 8.77	

*T0* preoperative, *T1* postoperative, *X* average of measurements, *SD* standard deviation

^*^Indicates a statistically significant difference. Surgical movement includes MA

The Group 2 patients with Angle Class II malocclusion who underwent MMA, showed significant post-surgical changes in the mCSA (*p* = 0.0005) and the TUAV(*p* = 0.01). The mCSA increased by an average of 83.13 mm^2^ (± 103.46), and the TUAV increased by an average of 5.44 cm^3^ (± 9.73). (Table 2). The average mandibular advancement was + 5.94 mm (± 3.25), and the average maxillary advancement was + 2.24 mm (± 2.42).

**Table 2:** Preoperative and postoperative mCSA and TUAV in subjects with Angle Class II facial deformity who underwent MMA

	mCSA (mm^2^)			TUAV (cm^3^)		
	X	SD	*p* Value	X	SD	*p* Value
T0	185.44	125.99		18.24	8.46	
T1	268.57	130.66	0.0005	23.68	10.92	0.01
Difference	(+)83.13	± 103.46		(+)5.44	± 9.73	

(+): Postoperative positive difference

Group 2 included two patients with diagnosed OSAS, both of whom underwent maxillomandibular advancement surgery. One patient received a maxillary advancement of 8.4 mm and a mandibular advancement of 9.2 mm. This resulted in an increase of 83 mm^2^ in the mCSA and a total airway volume increase of 4.3 cm^3^. The second patient underwent a maxillary advancement of 0.73 mm and a mandibular advancement of 6.45 mm. This led to an increase of 138.5 mm^2^ in the mCSA and a total airway volume increase of 9.8 cm^3^. During the postoperative follow-up period, the patients did not report any OSA-related symptoms.

The Group 3 patients with Angle Class III malocclusion underwent mandibular setback, and showed a decrease of 34.17 mm^2^ (± 107.47) in the mCSA and a decrease of 2.02 cm^3^ (± 8.81) in the TUAV; there were no significant changes in any airway measurements observed in this group (Table 3). The average mandibular setback was −4.16 mm (± 2.87).

**Table 3:** Preoperative and postoperative mCSA and TUAV in subjects with Angle Class III facial deformity who underwent mandibular setback

	mCSA (mm^2^)			TUAV (cm^3^)		
	X	SD	*p* Value	X	SD	*p* Value
T0	266.19	175.43		22.81	12.71	
T1	232.02	134.16	0.2228	20.79	7.68	0.3738
Difference	(+)34.17	± 107.47		(-)2.02	± 8.81	

Surgical movement mandibular setback. (-): Postoperative negative difference

The Group 4 patients who had Angle Class III malocclusion and underwent mandibular setback and maxillary advancement, did not show significant post-operative changes, with an average mCSA of 10.96 mm^2^ (± 111.52) and average total volume of 3.2 cm^3^ (± 8.32) (Table 4). The average mandibular setback was −3.97 mm (± 3.17), and the average maxillary advancement was 3.08 mm (± 1.76).

**Table 4:** Preoperative and postoperative mCSA and TUAV in subjects with Angle Class III facial deformity who underwent mandibular setback and maxillary advancement

	mCSA (mm^2^)			TUAV (cm^3^)		
	X	SD	*p* Value	X	SD	*p* Value
T0	298.82	125.08		24.10	8.99	
T1	309.78	149.06	0.6420	27.30	11.83	0.0793
Difference	(+)10.96	± 111.52		(+)3.19	± 8.32	

Following an average mandibular setback of −4.95 mm (±3.06) none of our patients in groups 3 and 4 developed OSA or exhibited any OSA-related symptoms during the postoperative follow-up period of one year.

The 95% confidence interval for the total volume (cm^3^) in prognathic patients who underwent either mandibular setback with or without a maxillary advancement was −1.57 to 4.87 (*p* = 0.40), and for the minimal cross-section area (mm^2^) was −39.56 to 29.10 (*p*=0.76), indicating in both parameters a statistically non-significant difference.

Both total volume (cm^3^) and minimal cross-sectional area (mm^2^) showed statistically significant differences in retrognathic patients treated with mandibular advancement or MMA, with 95% CIs of 2.34–8.36 (*p* < 0.001) and 54.14–124.16 (*p* < 0.0001), respectively.

## Discussion

Mandibular advancement with BSSO is a well-established treatment for retrognathism and has a concomitant beneficial effect on the upper airways [20–27]. This study demonstrated that patients who underwent mandibular advancement surgery (Group 1) showed significant post-surgical changes in the minimal cross-section area (mCSA) and total upper airway volume (TUAV), regardless of age or gender. With an average mandibular advancement of + 3.84 mm (± 1.83 SD), the mCSA increased by 36% (92.26 mm^2^), and the TUAV by 21% (4.91 cm^3^). The average airway volume increase per 1 mm mandibular advancement was 1,28 cm^3^. These findings align with previous studies [21–26]. For instance, Kochel et al. [24] reported a 46.9% increase in mCSA and 32% in TUAV, while De Oliveira et al. [25] observed a 6.9 cm^3^ increase in airway volume with combined mandibular advancement and genioplasty.

While MA employs beneficial techniques, MMA is considered to be the most effective surgical method for enlarging upper airway dimensions by expanding the skeletal framework, a technique commonly used to treat OSAS [24, 28–31]. According to Faria et al*.* [28] and Bianchi et al*.* [29], MMA creates more tension in the surrounding pharyngeal tissue and tongue, enhancing upper airway stability.

Veys et al. [30] observed a 35.4% (10.19 cm^3^) TUAV increase in OSAS patients following MMA. The mean maxillary advancement was 4.8 ± 2.8 mm, while the mean MA was 8.3 ± 2.3 mm. However, the study did not measure the pre- or postoperative changes in the mCSA. Likewise, Ravelo et al*.* [31] reported mCSA and TUAV increases of 28.14 mm2 and 8.4 cm3, respectively, following average maxillary and mandibular advancements of + 2.45 mm and + 4.25 mm. The results of our investigation were similar to those found in these studies [30, 31]. The patients who underwent MMA (Group 2) received an average MA of + 5.94 mm (± 3.25 SD), and an average maxillary advancement of + 2.24 mm (± 2.42 SD), and showed significant mCSA and TUAV increases of 83.13 mm^2^ (44.8%) and 5.44 cm^3^ (29.8%), respectively.

Mandibular setback surgery is a widely used treatment for patients with prognathism or skeletal Class III malocclusion, addressing both functional and esthetic concerns. While effective in correcting skeletal discrepancies, many studies have noted postoperative narrowing of the pharyngeal airway [16, 32–38]. Other investigations claim that bimaxillary surgery (i.e., maxillary advancement and mandibular setback) may contribute to a smaller reduction of the pharyngeal airway than isolated mandibular setback alone [35, 38].

Hong et al*.* [33] compared isolated mandibular setback surgery with bimaxillary surgery, revealing a significant narrowing in both groups. However, the decrease in the pharyngeal airway was smaller in the bimaxillary surgery group, compared to the isolated mandibular setback surgery group. Conversely, Y. Lee et al. [36] reported volumetric increases in the upper airway and decreases in the lower airway following bimaxillary surgery, though total volume changes were not statistically significant.

Havron et al. [37] found that isolated mandibular setback increased the minimum axial area, while bimaxillary surgery led to significant gains in airway volume and axial areas, including the retropalatal and retroglossal regions. They attributed this to anterior displacement of the tongue and soft tissues from maxillary advancement and concluded that mandibular setback can be safely performed without airway narrowing, though bimaxillary surgery may be preferable for borderline OSAS patients.

According to our observations, neither the TUAV nor the mCSA significantly changed following mandibular setback with or without maxillary advancement. Group 3 patients (Class III malocclusion) received an isolated mandibular setback with an average displacement of 4.16 mm (± 2.87), with non-significant decreases in the mCSA (34.17 mm^2^ ± 107.47 SD) and TUAV (2.02 cm^3^ ± 8.81 SD). Group 4 patients who underwent bimaxillary (mandibular setback and maxillary advancement) surgery showed postoperative increases in mCSA (mean 10.96 mm^2^, ± 111.52 SD) and TUAV (mean 3.2 cm^3^, ± 8.32 SD), with an average mandibular setback of −3.97 mm (± 3.17 SD) and maxillary advancement of 3.08 mm (± 1.76 SD). Notably, the mandibular setback in Group 3 was larger than in Group 4, and Group 4 showed an increase in the postoperative TUAV, while in Group 3, the same parameter was reduced. However, these changes in Group 4 were non-significant.

The potential role of surgical airway constriction in predisposing patients to OSAS remains controversial. Kobayashi et al*.* [38] noted initial decreases in oximetric indices following mandibular setback surgery, which normalized over six months. Obese subjects were more prone to persistent sleep-disordered breathing. Similarly, Engboonmeskul et al*.* [39] reported a postoperative airway space reduction without any occurrence of OSAS, though larger setbacks (> 6 mm) were associated with increased AHI and altered sleep architecture. Our data indicated that setbacks < 4.05 mm were not associated with significant TUAV reduction. Nonetheless, the risk factors of sleep-breathing disorder should be taken into account during the preoperative treatment planning stage to avoid the development of OSAS.

Furthermore, a direct correlation between the displacement distance and the degree of airway change is unclear. Although some authors reported a significant correlation between the extent of jaw movement and changes in airway measurements [33, 40, 41], others did not [13, 38, 42–44]. Our Spearman correlation analysis showed only weak associations between displacement distance and airway changes across all groups. This variability may be influenced by soft tissue elasticity, postoperative edema, hyoid bone repositioning, and body mass index (BMI) [45, 46].

Moreover, patients in our study with similar displacement distances exhibited variable airway outcomes, potentially due to the degree of the counter-clockwise mandibular rotation [23]. This rotation advances the mandibular symphysis and anteriorly displaces the tongue and suprahyoid muscles, thereby expanding the pharyngeal airway [5, 47]. Studies [27, 48] suggested that mandibular occlusal plane changes through counterclockwise rotation are important contributors to increasing the upper airway volume and that only sufficient rotation can treat and prevent postoperative residual OSAS [48]. The extent of airway change depends on the magnitude of counter-clockwise rotation, but individual variations, such as impaction and soft tissue or skeletal compensation, make it difficult to predict outcomes based on jaw displacement alone.

Notably, the results in Group 2 (mCSA *p* = 0.0005, total volume *p* = 0.01) were more significant than in Group 1 (mCSA *p* = 0.0048, total volume *p* = 0.0346). As the entire maxillomandibular complex is displaced in a bimaxillary surgery, a further widening of the airway and constriction areas is possible, subsequently increasing the total volume and mCSA values even further. Nonetheless, our results demonstrate that both MMA and an isolated MA surgery on patients with Class II malocclusion produce significant increases in the TUAV and mCSA. Isolated MA can be considered an effective surgical approach to treating oropharyngeal airway deficiencies, especially in cases where bimaxillary advancement cannot be performed. On the other hand, bimaxillary surgery should be contemplated when a further increase of mCSA and total airway volume is required.

The main goals of maxillary advancement in Class III dentofacial deformity cases are to establish a stable airway and dental occlusion, facial symmetry, and a reduced mandibular setback. Our results showed that a combined maxillary advancement may reduce the extent of mandibular setback while simultaneously achieving an enlarged posterior airway space. Furthermore, our investigation showed mandibular setback movements less than 4.05 mm were not related to a significant reduction in total airway volume.

Limitations of this study include the small sample size, short follow-up, and that an improvement or occurrence of clinical OSAS symptoms in the long term was not assessed. Although our results showed insignificant mCSA and TUAV decreases following isolated mandibular setback and confirmed MA and MMA as effective procedures to enlarge the upper airway dimensions, pre-and postoperative polysomnography was not performed to analyze patient sleep parameters (e.g. AHI). Although patients with BMI > 30 kg/m^2^ were excluded and most participants were within normal weight ranges, BMI variability within the non-obese range may still influence airway morphology and represents a limitation. Furthermore, the use of static CBCT imaging of the upper airway in an awake, upright sitting patient does not capture the dynamic physiology of the airway patency during sleep, such as soft tissue collapse or neuromuscular activity, limiting the assessment of functional airway behavior. Therefore, any conclusions regarding OSA or sleep-disordered breathing based solely on these static measurements should be interpreted with caution. Additionally, OSAS is a multifactorial disorder involving anatomical, neuromuscular, and systemic factors not fully addressed in this study. Finally, radiation exposure inherent to CBCT was minimized following the ALARA principle, which may restrict imaging frequency and timing, affecting longitudinal evaluation.

## Conclusions

This study emphasizes the importance of personalized surgical planning tailored to each patient’s unique characteristics. Our findings suggest that MA and MMA are effective surgical approaches for treating oropharyngeal airway deficiencies in Class II patients. Moreover, combining maxillary advancement may reduce the extent of mandibular setback while enlarging posterior airway space, and mandibular setbacks less than 4.05 mm do not cause significant total airway volume reductions in Class III patients. Studies should include a long follow-up period to examine long-term airway stability and the effects of orthognathic treatment on skeletal remodeling. The use of virtual planning tools holds great potential to improve the accuracy and individualization of orthognathic surgical patient care.

## Data Availability

The data are available upon request from the corresponding author.

## Abbreviations

MA: Mandibular advancement surgery
MMA: Maxillomandibular advancement surgery
CBCT: Cone beam computed tomography
mCSA: Minimal cross-section area
TUAV: Total upper airway volume
OSAS: Obstructive sleep apnea syndrome
BMI: Body-Mass-Index
LFI: LeFort I Osteotomy
BSSO: Bilateral sagittal split osteotomy
DICOM: Digital Imaging and Communications in Medicine
NHP: Neutral head position
X: Mean
SD: Standard deviation
REM: Rapid eye movement
AHI: Apnoea-hypopnoea index
